# Effects of back-support exoskeletons with different functional mechanisms on trunk muscle activity and kinematics

**DOI:** 10.1017/wtc.2023.5

**Published:** 2023-04-19

**Authors:** Benjamin Reimeir, Maité Calisti, Ronja Mittermeier, Lennart Ralfs, Robert Weidner

**Affiliations:** 1 Institute of Mechatronics, University of Innsbruck, Innsbruck, Austria; 2 Department of Sport Science, University of Innsbruck, Innsbruck, Austria; 3 Laboratory of Manufacturing Technology, Helmut-Schmidt-University/University of the Federal Armed Forces Hamburg, Hamburg, Germany

**Keywords:** Exoskeleton, support effect, biomechanical evaluation, muscle activity, range of motion, logistics

## Abstract

Musculoskeletal disorders constitute the leading work-related health issue. Mechanical loading of the lower back contributes as a major risk factor and is prevalent in many tasks performed in logistics. The study aimed to compare acute effects of exoskeletons with different functional mechanisms in a logistic task. Twelve young, healthy individuals participated in the study. Five exoskeletons with different functional mechanisms were tested in a logistic task, consisting of lifting, carrying, and lowering a 13 kg box. By using electromyography (EMG), mean muscle activities of four muscles in the trunk were analyzed. Additionally, kinematics by task completion time and range of motion (RoM) of the major joints and segments were investigated. A main effect was found for *Musculus erector spinae*, *Musculus multifidus*, and *Musculus latissimus dorsi* showing differences in muscle activity reductions between exoskeletons. Reduction in ES mean activity compared to baseline was primarily during lifting from ground level. The exoskeletons SoftExo Lift and Cray X also showed ES mean reduction during lowering the box. Prolonged task duration during the lifting phase was found for the exoskeletons BionicBack, SoftExo Lift, and Japet.W. Japet.W showed a trend in reducing hip RoM during that phase. SoftExo Lift caused a reduction in trunk flexion during the lifting phase. A stronger trunk inclination was only found during lifting from the table for the SoftExo Lift and the Cray X. In conclusion, muscle activity reductions by exoskeleton use should not be assessed without taking their designed force paths into account to correctly interpret the effects for long-term injury prevention.

## 1. Introduction

In workplaces in the European Union, musculoskeletal disorders (MSD) constitute the leading work-related health issue over the years and account for complaints by three out of five employees (Eurostat, 2010; European Agency for Safety and Health at Work, 2019). In absolute figures, around 40 million workers in European industrial workplaces suffer from MSD (Roquelaure, 2018). Among the types of MSD, backache and muscular pain have been found to be most prevalent (European Agency for Safety and Health at Work, 2019) and, in the example of the lower back region, affect 30% of the complaints (Eurostat, 2010). In the long term, work-related MSD cannot only cause strain and pain but are also reported to be the most common reason for sick leave of the workforce (Barthelme et al., 2021). In these cases, musculoskeletal complaints can also lead to a reduced quality of life at work or even to longer periods of absence (Roquelaure, 2018). For example, about a quarter of all cases lead to permanent disability at workplaces (Eurostat, 2010). The relevance of preventing MSD is gaining in importance, especially against the exacerbating factors, such as demographic change (European Agency for Safety and Health at Work, 2019; Barthelme et al., 2021) and increased working lifetime into older age.

One major cause of MSD is exposure to ergonomic risk factors since repeated exposure can exacerbate the problem (Barthelme et al., 2021). Specifically, as often occurring in industrial logistic tasks, performing in strenuous and awkward working postures (Barthelme et al., 2021) or repetitive movements (in 61% of cases), carrying or moving high load weights (32%), and working in tiring or painful postures (43%) are among the most common drivers of MSD (Eurostat, 2010; Eurofound, 2017; da Costa & Vieira, 2009). Accordingly, mechanical loading of the lower back (lumbar part of the spine) contributes as a risk factor (Roquelaure, 2018; European Agency for Safety and Health at Work, 2019).

In the past, workplace interventions to prevent MSD primarily focused on lowering biomechanical loads or implementing technical or organizational measures to limit workers’ exposure to intense or repetitive loads (Roquelaure, 2018). Against the background of high physical and also psychological stress in the workplace, the use of human-centered support is becoming increasingly important. As a more recent development, the use of exoskeletons in production and logistics has grown rapidly in the past few years (Weidner et al., 2020; Hoffmann et al., 2021). Exoskeletons are externally wearable mechanical systems designed to provide physical support of users (de Looze et al., 2016; Ralfs et al., 2021), helping the user perform movements or stabilize postures (Weidner and Hoffmann, 2020) and, thus, aiming to reduce workplace causes of MSD (Fox et al., 2019). Occupational exoskeletons can be classified as active and passive devices which are often also referred to as powered and nonpowered exoskeletons (de Looze et al., 2016; Fox et al., 2019; Crea et al., 2021). Passive exoskeletons use components (e.g., elastic bands, springs) to store and return energy through the user’s movements, whereas active exoskeletons use an external power supply to contribute linear force or torque to the user’s movements (e.g., electric or pneumatic drives) (de Looze et al., 2016; Weidner et al., 2020).

Common biomechanical and physiological methods to measure acute effects of exoskeletons are EMG, ergo spirometry, near-infrared spectrometry for tissue oxygenation, and motion capture to evaluate joint kinematics (Hoffmann et al., 2022). Focusing on studies of the lower back, different studies reveal occurring biomechanical and work-physiological effects for both active and passive exoskeletons. Nevertheless, it can be stated that the provided support by an exoskeleton may sometimes only lead to shifting loads to other bodily regions or muscular structures (Koopman et al., 2019). Regarding studies of EMG, several have shown a reduced activity of the trunk extensor muscles by using passive back exoskeletons (Bosch et al., 2016; Alemi et al., 2019, 2020; Koopman et al., 2019; Glitsch et al., 2020). Bosch et al. (2016) reported a decrease in trunk muscle activity of 35–37% while performing a static trunk bending and a static holding task with the Laevo exoskeleton. Similar results were found in Alemi et al. (2019), where a decreased activity of about 30% was found for the erector spinae longissimus muscle in a symmetric lifting task. For the use of active exoskeletons, Poliero et al. (2020) showed a reduction in lumbar activity by up to 12% and the greatest effect while carrying the heaviest load. Further, similar results were found by Kim et al. (2021) who demonstrated reduced muscle activity in the erector spinae (11% right side and 16% left side) using an active prototype exoskeleton. Regarding the effects of exoskeletons on kinematics, Poliero et al. (2020) found a reduction in hip and knee range of motion (RoM) of about 10%, and an increase in stride duration of 6–8% while using an active exoskeleton.

However, study designs are often inconsistent and only allow a limited general conclusion due to their focus on merely one exoskeleton. The investigated tasks are often limited to what the exoskeleton is specifically designed for (e.g., lifting an object, static forward-bending). Laboratory-based studies with strict movement instructions allow for a well-controlled environment to assure a clearer cause-effect relationship on the use of exoskeletons. Unfortunately, those findings are rarely transferable to real workplace applications. Based on existing studies using more complex logistic tasks (Baltrusch et al., 2018; Chen et al., 2018), this study considered a combined task in a laboratory environment. The main reason for this was that physically demanding jobs, such as those found in logistics often consist of a combination of different activities, such as lifting, carrying, walking, and lowering heavy loads. Further, a comparison of different exoskeletons allowed a more comprehensive conclusion on their functionality. The study design also tried to orientate on realistic applications, as it did not prescribe any standardized movements or procedures on how to perform each task. By doing so, it helps to gain valuable insights for the appropriate application of commercial exoskeletons in real workplace environments.

The aim of this study was to investigate the effects of three passive and two active exoskeletons with different functional principles on the activity of trunk muscles and body kinematics during a combined logistic activity. It was hypothesized that there will be a change in mean muscle activity (EMG), task completion time, and joint angle RoM between phases (such as lifting a box from the floor, carrying a box, placing the box on a table, lowering the box on the floor) and exoskeletons.

## 2. Methods

### 2.1. Participants

Twelve healthy students of the University of Innsbruck (three female, nine male, 27.2 ± 1.8 years, 75.3 ± 11.3 kg, 179.4 ± 9.2 cm) were included in the study. All participants were informed about the measurement and the potential risks, and provided written informed consent to participate prior to testing. The study was approved by the Board of Ethical Questions in Science of the University of Innsbruck on December 14, 2021 (Certificate 85/2021).

### 2.2. Experimental procedure

**Figure 1.**
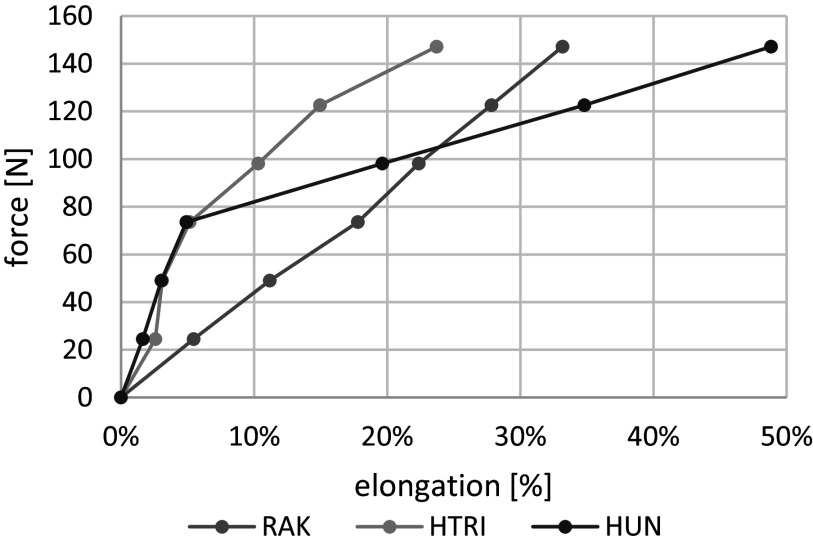
Spring force – elongation relationship of the three passive exoskeletons. Distinctive points in the profile of HUN can be seen at 5%, for HTRI at 5 and 15% elongation.

The data used in this study was extracted from a dataset collected between November 21, 2021 and February 28, 2022 in the biomechanical laboratory at the Chair for Production Technology at the University of Innsbruck. The whole dataset consists of nine different tasks performed by volunteers in the industrial test course for the evaluation of exoskeletons designed by Ralfs et al. (2021) using two active back-support exoskeletons (Japet’s Japet.W – JAP and German Bionic’s Cray X – CRAY) and three passive exoskeletons (HUNIC’s SoftExo Lift – HUN, N-Ippin’s Rakunie – RAK, and hTRIUS’ BionicBack – HTRI) (see Table 1 and Figure 1). Additionally, exoskeletons supporting the upper extremities were tested in this data collection. Due to the duration and volume of measurements, the data collection was separated into two measurement sessions (~2 hr per session) to mitigate effects of fatigue. A baseline measurement without an exoskeleton was performed on both occasions.

**Table 1.** Relevant characteristics and specifications of the five exoskeletons tested in this study

Name of exoskeleton/manufacturer	Rakunie N-Ippin	BionicBack hTRIUS	SoftExo Lift HUNIC	Japet.W Japet	Cray X German Bionic
Type of mechanism	Passive	Passive	Passive	Active	Active
	Shoulder straps	Shoulder straps	Shoulder straps	Velcro-fastened belt around torso below sternum	Shoulder straps/chest vest
Upper/lower exoskeleton interface	Textile straps below knee on tibial tuberosity	Textile straps at middle position of thighs	Textile cuff around knees and ankles	Velcro-fastened belt around pelvic iliac crest	Cuffs at proximal position of the thighs
Material	Textile/soft	Back structure – PVC straps – textile/soft	Back structure – PVC, carbon straps – textile/soft	Textile/soft actuators – electric motors	Back structure –carbon, PVC, textiles thigh cuffs – PVC, textile/soft
Weight	250 g	1,280 g	950–1,150 g	2 kg	7 kg
Functional mechanism	Elastic elements along backside of body	Elastic elements from back structure to lower interfaces	Elastic elements from back structure to knee cuff	Lumbar traction by elongation of linear actuators	Torque around hip joint by rotary actuators
Technical details	Spring stiffness 440 N/m	Spring stiffness elongation <5% 1,430 N/m 5–15% 530 N/m >15% 320 N/m	Spring stiffness elongation <5% 1,500 N/m >5% 175 N/m	Linear actuators 4 × 40 N	Rotary actuators 2 × 60 Nm
Force path	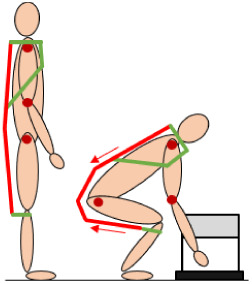	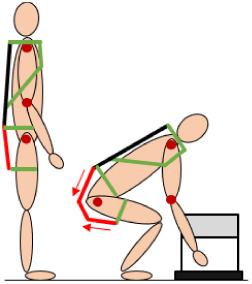	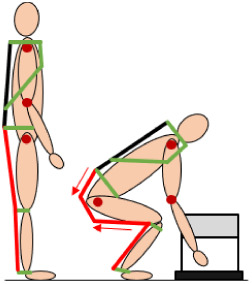	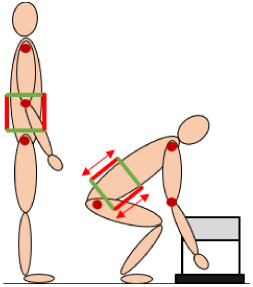	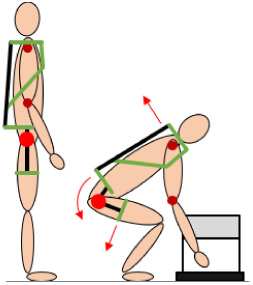
Picture	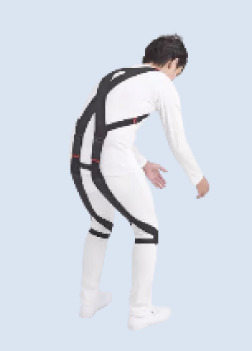	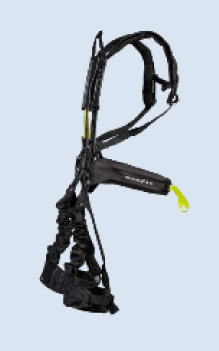	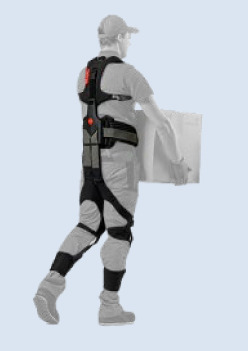	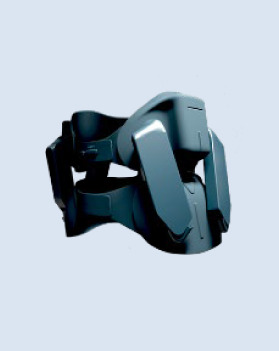	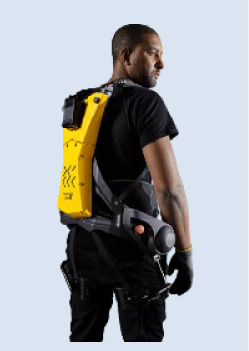

**Figure 2.**
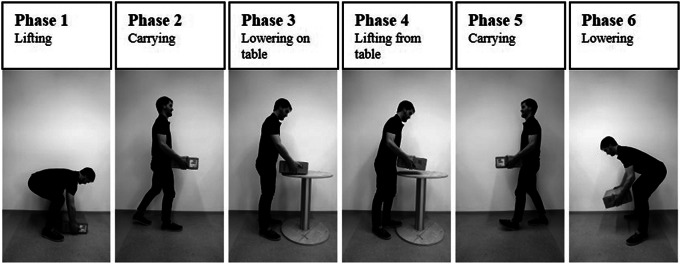
The separation of the combined logistic task in six phases based on the different movements.

From the nine different tasks, a combined logistic task simulated in a laboratory setting was used. Participants were instructed to lift a 13 kg box (37 × 37 × 15 cm) from the floor, carry the box 3 m and put it on an 84 cm high table, then pause for a moment, further pick up the box, turn around, walk back to the starting position, and finally lower the box down to the floor (Figure 2). Each participant performed the logistics task in six conditions – without any supportive device (baseline – BL) and with the five different exoskeletons. Two repetitions were performed for each support condition. The exoskeletons were donned with the assistance of the researchers according to the manufacturer’s guidelines and recommendations to assure a correct fit. A familiarization period of 5 min was carried out for each exoskeleton before testing, where participants could perform basic movements and adjust the settings to improve fit.

### 2.3. Instrumentation

An eight-channel wireless surface EMG system (Myon, Aktos, 2,000 Hz) was used for muscle activity recordings. Two pre-gelled Ag/AgCl electrodes (Ambu BlueSensor) with an electrode size of 10 mm and an interelectrode distance of 20 mm were placed in the direction of the muscle fibers on the prominent muscle bellies (Hermens et al., 2000). The activity of the following muscles was recorded in accordance with the SENIAM guidelines: *M. latissimus dorsi* (LAT), *M. erector spinae* (ES), *M. multifidus* (MF), and *M. obliquus externus abdominis* (OEA) of the dominant side (Hermens et al., 1999). For good electrode-skin contact, the area of the respective muscles was shaved, abraded, and then cleaned with an isopropyl alcohol swab to allow a low (<5 kΩ) cutaneous impedance of the skin. The sensors were protected with a 3D-printed cover and fixed with tape to minimize the pressure and movement from the exoskeletons’ waist bands. Maximal voluntary contractions (MVC) for the four muscles were measured on both occasions (Konrad, 2005). Contraction times were 5 s. 3D motion capture was measured with an inertial sensor tracking system (Xsens MVC Awinda system, 60 Hz). The participants were asked to wear inertial measurement units (IMUs) by using velcro straps and a Lycra T-shirt. According to the instructions given by Xsens, the 17 IMU sensors were attached to the back of the head, pelvis, sternum, and bilaterally to the shoulders, upper arms, forearms, hands, upper legs, lower legs, and feet. A calibration was carried out in accordance with the guidelines provided by Xsens. EMG recording and motion capture were time-synchronized via the Xsens MVN Analyze Pro 2021.0.1.

### 2.4. Data analysis

Flexion angles for ankle, knee, hip, shoulder joints, and the trunk using the ZXY Euler sequence were calculated in MVN Analyze Pro. In a custom-written Matlab program, the trials were separated into six consecutive phases, based on the activities performed in the combined logistic task (see Figure 2).

The separation of the phases was carried out based on distinctive kinematic parameters like hip angles and angular velocities (for lifting and lowering the box to the floor) and shoulder angles (placing and lifting the box from the table).

Raw EMG data were first filtered with a fifth-order high-pass Butterworth filter with a cut-off frequency of 30 Hz. Data were then fully wave-rectified and further filtered with a fifth-order low-pass Butterworth filter with a cut-off frequency of 2.5 Hz. EMG data of the four muscles were normalized to the maximum activity obtained from the moving average of the MVC trials. For further analysis, the mean and peak EMG amplitude over each previously described phase was taken for each condition (BL, RAK, HTRI, HUN, JAP, CRAY).

**Figure 3.**
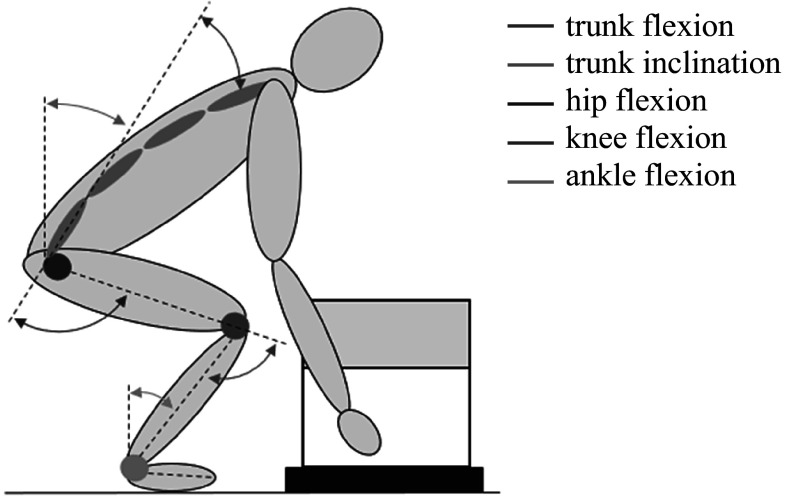
Joint angle conventions used in this study. Trunk inclination was calculated as tilt of pelvis relative to vertical axis. Trunk flexion was calculated between pelvis and T8.

For the kinematic analysis, the RoM for the ankle, knee, and hip joints (left and right) were calculated for each phase of the task as maximal value minus start and end value for lifting and placing the box down, and maximum minus minimum for the remaining phases (carrying, placing down, and lifting the box from the table), see Figure 3.

For both, kinematic and electromyographic analysis, the average of two repetitions was calculated. Differences (∆mean EMG, ∆RoM, ∆task duration) between exoskeletons and the respective baseline were calculated, as the order of the exoskeletons was randomized. For one participant, data obtained with one exoskeleton were excluded due to sensor displacement and lost signal. For two female participants, the data on active exoskeleton JAP was excluded due to misfit.

### 2.5. Statistical analysis

Statistical analyses were performed using IBM SPSS Statistics 27.0. The significance level was set at *p* < .05. Results with *p*-values above the significance level, but below 0.1 were mentioned as statistical trends. Data are presented as means and standard deviation (SD). Normal distribution was checked using the Shapiro–Wilk test. Further, a linear mixed model analysis of variance was performed because of the missing data as mentioned above. Exoskeletons and phases were considered as repeated factors, while dependent variables were either mean EMG, RoM, or task duration. Paired *t*-tests using Bonferroni correction of the alpha level, were conducted as posthoc tests to identify pairwise differences.

## 3. Results

**Figure 4.**
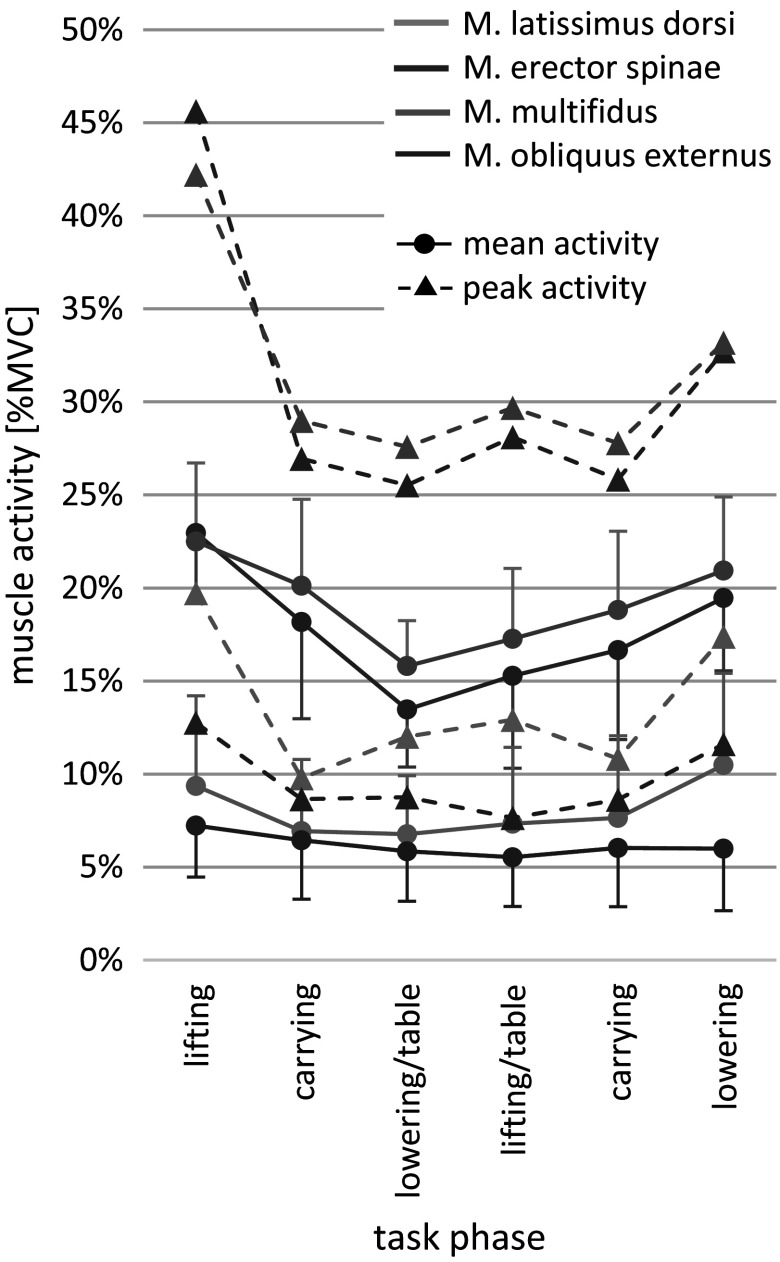
Mean and peak muscle activity as %MVC over each phase without exoskeleton support. Mean activity data are presented as mean ± 2SE. *n* = 12.

The combined task showed different muscle activations over the distinct phases. Especially phase 1 (lifting box from floor) and phase 6 (lowering box to floor) had notably higher mean and peak activations for LAT, ES, and MF compared to the other phases (see Figure 4).

Also, the effect of exoskeletons on muscle activity, task duration, and RoM varied between the phases. The main effect of phases was significant for ES (*F*(5, 82.378) = 7.022, *p* < .001, *η*^2^ = 0.299), LAT (*F*(5, 99.673) = 6.425, *p* < .001, *η*^2^ = 0.244), task time prolongation (*F*(5, 77.660) = 11.864, *p* < .001, *η*^2^ = 0.433) (see Table 2), and RoM in hip (*F*(5, 107.937) = 4.475, *p* < .001, *η*^2^ = 0.173) (see Table 2). A higher ES mean activity reduction through exoskeletons could be seen in phase 1 (lifting box from floor) compared to phase 2/5 (walking /carrying box) and phase 3 (placing box on table). Additionally, the exoskeletons’ effects were significantly stronger on LAT mean activity during phase 6 (lowering box down to floor) compared to phase 2/5 (walking/carrying box) and phase 3 (placing box on table). The duration to execute the tasks was significantly prolonged by the exoskeletons for phases 1 (lifting box from floor) and 6 (lowering box to floor) (see Figure 4).

**Table 2.** Statistical results of the main effects of phase and exoskeleton for all kinematic variables. n=12

				Phase 1: Lifting						Phase 2: Carrying						Phase 3: Lowering on table					
		Statistics		RoM [°]	Abs. change to BL [°]					RoM [°]	Abs. change to BL [°]					RoM [°]	Abs. change to BL [°]				
		Exo	Phase	BL	RAK	HTRI	HUN	JAP	CRAY	BL	RAK	HTRI	HUN	JAP	CRAY	BL	RAK	HTRI	HUN	JAP	CRAY
Kinematics	Ankle R	F(4,84.4) =.859 p =.492	F(5,114.4) =3.754 p =.003	27.4 ±5.2	0.8 ±7.9	0.5 ±10.5	0.8 ±10.1	−0.1 ±7.4	−0.3 ±6.0	40.6 ±4.5	−0.9 ±2.3	−0.6 ±3.0	−2.4 ±4.1	−0.7 ±2.6	−1.2 ±2.4	22.2 ±4.9	2.0 ±6.0	2.7 ±6.1	0.5 ±8.7	3.0 ±8.9	0.6 ±6.7
	Ankle L	F(4,94.3) =.1.977 p =.104	F(5,115.7) =1.832 p =.112	27.6 ±6.8	−1.1 ±6.8	1.5 ±8.9	−0.8 ±9.3	0.1 ±9.8	1.5 ±6.8	40.5 ±5.5	0.1 ±4.1	0.8 ±3.0	−2.4 ±4.8	0.4 ±3.6	0.6 ±2.4	22.3 ±7.4	−0.6 ±9.3	−1.3 ±7.5	−4.4 ±8.5	−0.3 ±8.9	0.8 ±7.5
	Knee R	F(4,49.5) =.702 p =.594	F(5,101.5) =5.942 p <.001	112.8 ±27.1	−12.5 ±16.9	−0.4 ±25.9	−8.5 ±21.5	0.8 ±27.1	−6.6 ±11.9	72.2 ±3.2	−0.3 ±2.7	−0.4 ±3.0	−1.1 ±5.1	0.5 ±3.9	1.0 ±4.2	44.1 ±14.6	1.1 ±3.7	1.5 ±3.6	−1.1 ±5.1	2.3 ±20.4	1.0 ±4.2
	Knee L	F(4,51.5) =2.722 p =.039	F(5,76.0) =5.318 p <.001	114.6 ±27.6	−12.2 ±13.8	−0.9 ±27.6	−10.1 ±21.9	3.1 ±26.8	−6.0 ±12.5	71.9 ±4.1	−1.1 ±2.4	0.3 ±2.5	−3.4* ±3.4	−3.1 ±4.7	1.1 ±4.3	36.6 ±13.8	0.0 ±4.3	0.9 ±3.1	−2.9 ±4.7	−1.8 ±5.3	1.4 ±3.7
	Hip R	F(4,111.2) =.256 p =.905	F(5,107.9) =4.475 p <.001	119.0 ±9.0	−4.2 ±5.7	−3.1 ±7.9	2.5 ±9.0	−10.5 ±11.5	−0.9 ±7.5	42.7 ±3.8	0.8 ±7.9	0.5 ±10.5	0.8 ±10.1	−0.1 ±7.4	−0.3 ±6.0	25.3 ±6.8	−0.9 ±2.3	−0.6 ±3.0	−2.4 ±4.1	−0.7 ±2.6	−1.2 ±2.4
	Hip L	F(4,97.3) =.987 p =.418	F(5,103.4) =2.159 p =.064	121.1 ±9.1	−5.0 ±6.5	−3.4 ±8.7	2.2 ±9.1	−10.4 ±11.6	−0.9 ±9.7	43.9 ±4.7	−1.1 ±6.8	1.5 ±8.9	−0.8 ±9.3	0.1 ±9.8	1.5 ±6.8	25.7 ±6.1	0.1 ±4.1	0.8 ±3.0	−2.4 ±4.8	0.4 ±3.6	0.6 ±2.4
	Trunk inclination	F(4,78.9) =3.832 p =.007	F(5,69.6) =1.513 p =.197	50.2 ±14.3	7.2 ±9.8	2.7 ±12.0	9.6 ±14.9	−1.4 ±13.0	6.4 ±8.6	6.5 ±4.8	−0.6 ±9.8	3.5 ±6.1	0.1 ±4.1	1.1 ±8.1	7.7 ±9.8	14.3 ±6.0	1.6 ±6.8	2.1 ±8.1	3.8 ±5.4	3.6 ±10.0	4.5 ±9.7
	Trunk flexion	F(4,101.1) =7.605 p <.001	F(5, 76.8) =0.374 p =.865	41.8 ±6.1	0.3 ±6.4	−2.0 ±7.1	−9.3* ±10.1	−5.6 ±9.7	−4.9 ±11.6	8.7 ±5.2	−2.0 ±7.0	−1.9 ±6.4	−2.9 ±6.6	−2.1 ±9.7	−4.4 ±7.6	12.2 ±6.4	−0.0 ±6.7	−3.2 ±6.4	−5.5 ±6.3	−4.9 ±8.2	−6.0 ±9.6
		Statistics		Time [s]	Abs. change to BL [s]					Time [s]	Abs. change to BL [s]					Time [s]	Abs. change to BL [s]				
Task duration		F(4,114.9) =5.201 p <.001	F(5,77.6) =11.864 p <.001	3.0 ±0.3	0.1 ±0.3	0.4* ±0.1	0.4* ±0.4	0.9* ±0.8	0.3 ±0.5	3.1 ±0.3	−0.2 ±0.6	0.1 ±0.4	−0.3 ±0.7	−0.0 ±0.5	0.1 ±0.4	2.1 ±0.3	−0.0 ±0.3	−0.0 ±0.3	0.1 ±0.3	0.1 ±0.1	−0.0 ±0.3

				Phase 4: Lifting from table						Phase 5: Carrying						Phase 6: Lowering on floor					
		Statistics		RoM [°]	Abs. change to BL [°]					RoM [°]	Abs. change to BL [°]					RoM [°]	Abs. change to BL [°]				
		Exo	Phase	BL	RAK	HTRI	HUN	JAP	CRAY	BL	RAK	HTRI	HUN	JAP	CRAY	BL	RAK	HTRI	HUN	JAP	CRAY
Kinematics	Ankle R	F(4,84.4) =.859 p =.492	F(5,114.4) =3.754 p =.003	16.2 ±4.2	2.1 ±7.5	1.4 ±5.7	2.3 ±5.5	2.2 ±5.2	−1.2 ±9.4	41.6 ±5.3	3.1 ±8.5	−0.4 ±6.9	−1.6 ±10.0	1.8 ±9.0	2.8 ±6.3	24.2 ±6.5	−0.4 ±2.7	−1.3 ±1.7	−4.0 ±4.6	0.1 ±2.7	−0.2 ±3.0
	Ankle L	F(4,94.3) =.1.977 p =.104	F(5,115.7) =1.832 p =.112	13.5 ±4.5	−1.8 ±12.9	−1.9 ±13.1	−2.8 ±12.9	0.2 ±10.0	0.9 ±11.2	38.9 ±5.4	0.2 ±5.9	2.3 ±9.9	−1.1 ±9.2	2.8 ±4.3	1.4 ±5.3	25.3 ±7.5	−0.7 ±1.8	−1.0 ±4.7	−4.2 ±5.0	−3.0 ±6.8	0.2 ±2.3
	Knee R	F(4,49.5) =.702 p =.594	F(5,101.5) =5.942 p <.001	28.8 ±8.4	6.2 ±14.3	4.0 ±21.3	9.0 ±19.2	2.3 ±20.4	−6.3 ±25.9	71.9 ±3.6	−0.2 ±5.3	2.7 5.2	1.8 ±5.8	6.4* ±6.5	3.7 ±6.1	105.0 ±27.6	−0.4 ±6.5	2.4 ±4.5	0.4 ±4.3	−0.5 ±6.5	−0.9 ±5.1
	Knee L	F(4,51.5) =2.722 p =.039	F(5,76.0) =5.318 p <.001	26.5 ±9.3	−3.4 ±24.9	−6.4 ±28.3	−9.3 ±17.1	5.7 ±26.7	11.2 ±26.3	71.5 ±4.1	0.8 ±6.0	−0.8 ±3.5	0.8 ±6.7	3. ±6.	3.4 ±6.5	104.7 ±27.7	1.7 ±5.1	4.3 ±7.2	1.8 ±3.2	2.1 ±4.4	0.6 ±3.9
	Hip R	F(4,111.17) =.256 p =.905	F(5,107.9) =4.475 p <.001	24.7 ±7.0	2.0 ±6.0	2.7 ±6.1	0.5 ±8.7	3.0 ±8.9	0.6 ±6.7	40.7 ±3.4	2.1 ±7.5	1.4 ±5.7	2.3 ±5.5	2.2 ±5.2	−1.1 ±9.4	116.4 ±10.4	3.1 ±7.9	−0.4 ±6.9	−1.6 ±10.0	1.8 ±9.0	2.8 ±6.3
	Hip L	F(4,97.3) =.987 p =.418	F(5,103.4) =2.159 p =.064	23.2 ±5.3	−0.6 ±9.3	−1.3 ±7.5	−4.4 ±8.5	−0.3 ±8.9	0.8 ±7.5	41.5 ±3.1	−1.8 ±12.9	−1.9 ±13.1	−2.8 ±12.9	0.2 ±10.0	0.9 ±11.2	116.6 ±9.0	0.2 ±6.0	2.3 ±9.9	−1.1 ±9.2	2.8 ±4.3	1.4 ±5.3
	Trunk inclination	F(4,78.9) =3.832 p =.007	F(5,69.6) =1.513 p =.197	15.1 ±5.6	0.7 ±7.0	3.1 ±6.4	5.4* ±5.1	6.3 ±9.5	8.3* ±8.6	4.4 ±5.2	0.4 ±7.7	2.4 ±5.7	1.6 ±3.9	2.5 ±7.7	6.3 ±9.3	46.0 ±16.1	5.6 ±11.9	4.7 ±14.4	11.8 ±15. 4	−0.8 ±16.8	11.1 ±12.9
	Trunk flexion	F(4,101.1) =7.605 p <.001	F(5, 76.8) =0.374 p =.865	14.4 ±7.4	0.3 ±7.2	−2.7 ±7.6	−6.8 ±10.1	−6.4 ±8.2	−5.1 ±9.3	9.3 ±6.1	−0.0 ±6.6	−1.6 ±7.1	−3.8 ±6.4	−5.1 ±8.8	−5.2 ±7.7	37.9 ±7.9	1.0 ±7.7	−1-5 ±8.8	−9.0 ±10.4	−4.1 ±10.6	−4.3 ±11.9
		Statistics		Time [s]	Abs. change to BL [s]					Time [s]	Abs. change to BL [s]					Time [s]	Abs. change to BL [s]				
Task duration		F(4,114.9) =5.201 p <.001	F(5,77.6) =11.864 p < .001	2.5 ±0.3	−0.2* ±0.1	0.0 ±0.4	0.1 ±0.4	0.3 ±0.9	−0.2 ±0.4	2.9 ±0.3	0.0 ±0.2	−0.3 ±0.4	−0.4 ±0.6	−0.0 ±0.6	0.1 ±0.4	3.1 ±0.5	−0.1 ±0.4	0.3 ±0.4	0.4 ±0.6	0.7* ±0.5	0.1 ±0.3

*Note.* BL values and absolute differences to BL of RoM for all investigated joints and task durations separated into the six phases. The values are presented as mean ± SD. Asterisks (*) indicate statistically significant differences compared to BL.

As presented in Table 2, hip RoM was significantly reduced by the exoskeletons during phase 1 (lifting box from floor) and phase 3 (placing box on table) compared to baseline.

### 3.1. Combined task

The statistical analysis showed a significant fixed effect for exoskeletons in the mean activity of ES (*F*(4, 82.270) = 8.90, *p* < .001, *η* ^2^ = 0.302), MF (*F*(4, 112.710) = 6.891, *p* < .001, *η* ^2^ = 0.197), and LAT (*F*(4, 116.135) = 12.598, *p* < .001, *η* ^2^ = 0.302). When considering the overall combined task, the relative reduction in mean ES activity compared to BL was not significantly different between HTRI (−14%), HUN (−12.8%), and CRAY (−14.5%). Similar results were found for MF, where HTRI showed the biggest relative reduction in mean activity (−18.9%), followed by HUN (−8.1%), and CRAY (−9.8%). For LAT, reduced activity compared to baseline was found for HTRI (−11.8%), HUN (−12.6%), JAP (−15.2%), and CRAY (−24.2%). No significant reduction in mean muscle activity could be seen for the exoskeleton RAK. The exoskeletons showed no effect on the mean muscle activity of OE in the combined tasks (*F*(4, 47.662) = 1.036, *p* = .398, *η* ^2^ = 0.080).

### 3.2. Task separated in phases

**Figure 5.**
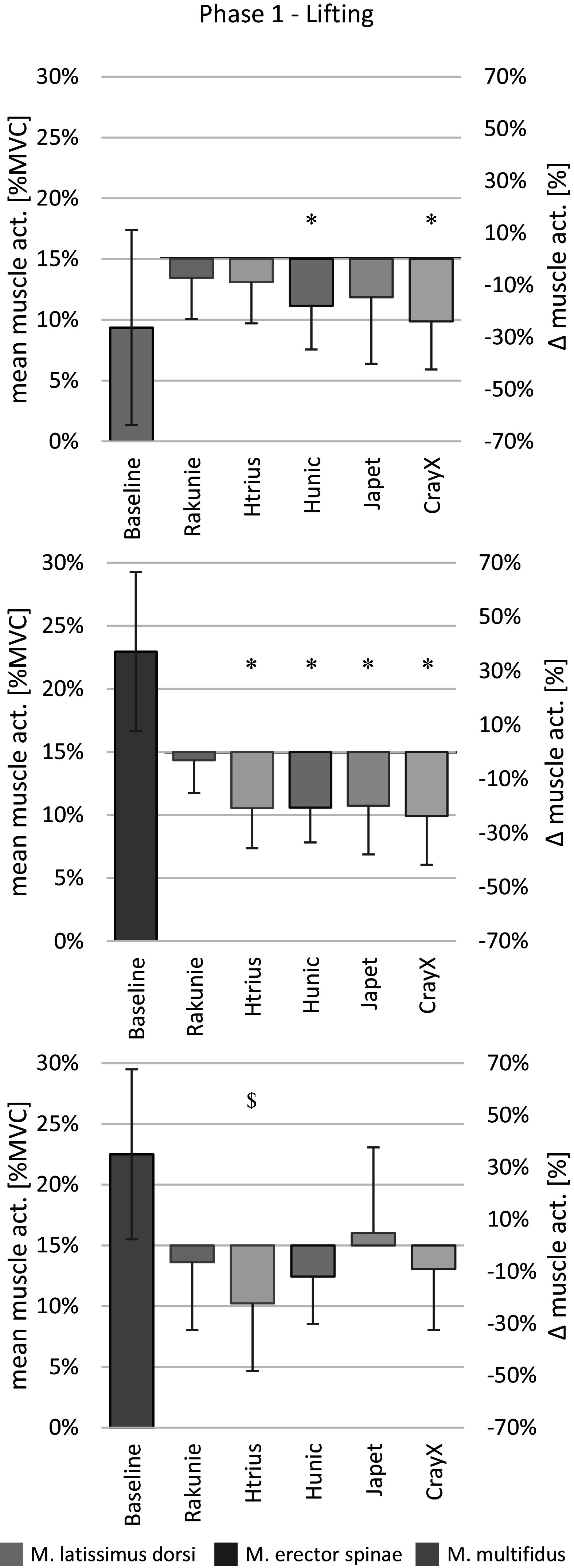
Mean muscle activity during the lifting of the box from the floor as %MVC and relative change due to exoskeleton support. Data are shown as mean ± SD. *p < .05; ^$^*p* < .1. n=12

**Figure 6.**
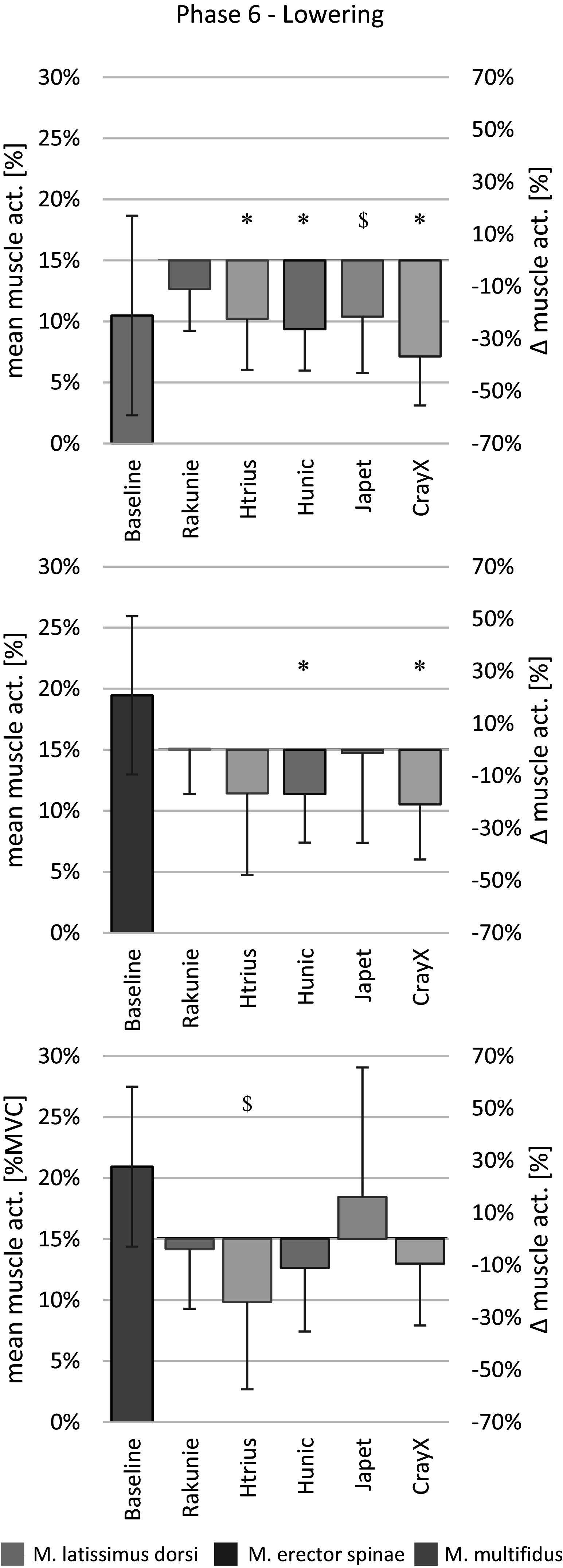
Mean muscle activity during the lowering of the box to the floor as %MVC and relative change due to exoskeleton support. Data are shown as mean ± SD. *p < .05; ^$^*p* < .1. n=12

The following results were found when separating the overall combined task into the different phases, focusing on phase 1 (lifting box from floor) and phase 6 (lowering box down to floor) (see Figures 5 and 6). These phases showed the highest mean and peak muscle activities during baseline measurement, and also the strongest effects in absolute reduction of mean activity by the exoskeletons.

#### 3.2.1. Phase 1: Lifting box from floor

Absolute mean EMG activity for LAT, ES, and MF during phase 1 (lifting box from floor), and the relative reduction by each exoskeleton are shown in Figure 5. HUN and CRAY caused a significant reduction in LAT mean activity during phase 1, which was 18.0 and 24.0%, respectively. On average, CRAY showed the highest reduction in ES mean activity during the lifting of the box with 23.7%. The use of HTRI, HUN, and JAP caused similar EMG reductions of about 20%. The reduction in MF mean activity did not significantly differ between the separate phases. HTRI showed a mean activity reduction of 22.3% in phase 1. RAK and JAP showed no significant effect on MF mean activity.

The duration of phase 1 was significantly prolonged by HTRI (0.36 s; 12%), HUN (0.40 s; 13%), and JAP (0.92 s; 30%) (see Table 2). The use of CRAY showed a statistical trend in slowing down the lifting from the floor by 0.34 s (11%) on average. The use of RAK did not influence the duration of phase 1.

Regarding the influence of the individual exoskeletons on the RoM in hip, knee, and ankle joints during phase 1, no relevant statistically significant effects were observed, although some trend can be seen. JAP reduced hip RoM on average by 10° (right: *p* = .069; left: *p* = .064), as presented in Table 2. Knee RoM was reduced by wearing the exoskeleton RAK by about 12° (right: *p* = .132; left: *p* = .059) (see Table 2). Further, the passive exoskeleton HUN showed a significant reduction in trunk flexion of 9.3° (*p* = .047) (see Table 2). No significant effect was observed for the variable trunk inclination (see Table 2).

#### 3.2.2. Phase 6: Lowering box to floor

Figure 6 displays the absolute mean muscle activities for LAT, ES, and MF during phase 6 (lowering box to floor) and the relative reduction by each exoskeleton. Similar to phase 1, CRAY showed the highest significant mean activity reduction in LAT and ES with 36.7 and 20.9%, respectively. JAP and RAK did not cause a significant change in muscle activity for LAT, ES, and MF while lowering the box to the floor. The reduction in MF mean activity caused by HTRI was not significant after alpha-level correction but showed a statistical trend with an average reduction of 24.0%.

JAP significantly prolonged the duration of phase 6 by 0.65 s (21%). The other exoskeletons showed no significant effect on task duration, even though a trend for HTRI (+0.32 s, 10%) and HUN (+0.40s, 13%) can be recognized (see Table 2).

A reduction in ankle RoM was observed during phase 6 (lowering box to floor) for the exoskeleton HUN by about 4° (R: *p* = .064; L: *p* = .069). It needs to be mentioned that it was not found to be statistically significant after alpha-level correction. No other relevant effects on the RoM of the investigated major body joints were observed during phase 6 (lowering box to floor), neither for trunk flexion, nor trunk inclination (see Table 2).

#### 3.2.3. Phases 2–5: Walking/carrying box, lowering, and lifting box from table

Notable is the reduction in LAT mean activity during these phases by the active exoskeletons JAP and CRAY, where the latter one outperformed the other exoskeletons regarding the reduction of LAT activity in all phases. Some smaller relative reductions in ES mean activity during phases 2–5 were found for the exoskeletons HUN and CRAY.

Neither task duration nor RoM in hip, knee, and ankle, nor trunk flexion during carrying, lowering, and lifting the box from the table were significantly affected by any exoskeleton (see Table 2). However, a significant increase in trunk inclination was found for the exoskeleton HUN with 5.4° (*p* = .024) and the exoskeleton CRAY with 8.3° (*p* = .044) in phase 4, lifting box from table (see Table 2).

## 4. Discussion

The aim of this study was to investigate the effect of exoskeletons with different functional principles on the activity of back and trunk muscles, and the kinematics of trunk and body kinematics during a combined logistic working task.

### 4.1. Influence of exoskeleton use on muscle activity

In general, the use of an exoskeleton in this study led to a decrease in muscle activity of the back muscles. This reduction varied between the different exoskeletons and the separated phases. The effect of the exoskeletons on ES mean activity was the highest during the lifting of the box from the floor (phase 1). HTRI, HUN, JAP, and CRAY showed similar activity reductions with 20–24%. On the contrary, LAT activity was reduced most during the lowering of the box to the floor (phase 6), where CRAY showed the highest reduction with over 35% on average. During that phase, the effect of JAP on ES mean activity was diminished. The relative reduction of the MF muscle activity was not significantly changing over the separate phases and was highest for the HTRI exoskeleton. JAP led to no significant change in MF muscle activity. RAK caused no significant effect on the activity of any of the observed muscles. The mean activity of the abdominal muscles was not affected by the use of an exoskeleton.

This is in part similar to previous findings, mainly concerning the effects of passive lifting-assistive devices during static posture or regulated lifting movements. Most studies focused on the effects on the iliocostalis and longissimus part of the ES muscle, reporting relative reductions in peak and mean activity between 14 and 35%, strongly depending on load and lifting style (Abdoli-E et al., 2006; Frost et al., 2009; Lamers et al., 2017; Alemi et al., 2019; Glitsch et al., 2020). Bosch et al. (2016) found an even higher relative reduction for upper back muscles (44%) than for the lower back muscles (35–38%) during a forward-bent assembly task wearing a Laevo exoskeleton.

This exoskeleton creates a torque around the hip joints with interfaces at the chest and the front of the thighs. Compared to this study, the EMG reductions we found were generally lower. This might be due to the dynamic characteristics of the executed task in this study. But, similar to Bosch et al. (2016), the muscle activity reductions in the upper back muscles during the lowering phase, especially using CRAY (37% mean act. red.), were also higher compared to the lower back muscles (21% mean act. red.).

When considered in isolation, ES and MF function as extensors of the lumbar spine, the latter especially with regards to the lower segments (L4/L5 and L5/S1) (Kendall et al., 2005). However, in heavy lifting tasks, stability of the trunk is regarded as a major contributor to the health and safety of the spinal structure (Zazulak and Medvecky, 2019). Biomechanists tend to refuse to separate functions of single muscles in the trunk in the context of intraabdominal pressure and trunk stability (McGill et al., 2003; Hodges et al., 2005). Hence, we also consider ES, MF, and OE activity in their synergistic function of stabilizing the trunk. Studies also mention LAT in this regard as a synergistic support for extension of the lumbar spine (Kendall et al., 2005) and biomechanical simulations support these findings (Cholewicki and Vanvliet IV, 2002). Even though the isolated biomechanical function and role of LAT may only have minor effects on spinal extension directly (McGill and Norman, 1988), the imposed load by the carried box has to be transmitted from the upper extremities to the trunk and the lower body in the investigated lifting task.

Concerning the abdominal muscles, the majority of studies reported no significant changes in muscle activity elicited by the use of exoskeletons (Abdoli-E et al., 2008; Baltrusch et al., 2020). In contrast to that, Frost et al. (2009) modulated the elastic stiffness of a PLAD to optimize relieving effects for the back, leading to high reductions in muscle activity of the lumbar and thoracic back muscles but also a notable increase in OE activity by 138% during stoop lifting.

### 4.2. The role of exoskeleton design and functional mechanism

#### 4.2.1. Passive exoskeletons

Elastic stiffness, material, and functional mechanism of the exoskeletons play an essential role not only in the reduction or regional shift of muscle activity (Barret and Fathallah, 2001), but more importantly in the biomechanical and neurophysiological causes eliciting the altered activation signal. Most studies remain superficial in their analysis of muscle activity reduction, and only more recently, authors have begun conducting an in-depth analysis of the functional mechanical principles of the support devices (Näf et al., 2018; Koopman et al., 2019; Glitsch et al., 2020; Luger et al., 2021).

The function of the three passive exoskeletons used in this study (RAK, HTRI, HUN) is based on elastic elements running alongside the posterior chain (see Table 1). Depending on the position of the elastic elements and the interface (e.g., at the thighs, below the knee, at knee and ankle), a combination of hip and trunk flexion leads to the desired supportive function of the exoskeleton. According to Hill’s muscle model, this mechanism mainly redistributes applied forces from the contractile elements (back and hip extensor muscles) to the external parallel elastic elements of the exoskeleton (Hill, 1938, 1950). Therefore, we argue that the supportive effect concerning the mechanical loads on the passive structures of the spine is minimal for an exoskeleton design based on this functional principle.

#### 4.2.2. Lumbar traction and interface position

Effects of exoskeletons on the activity of the lower back muscles (e.g., MF) are rarely reported in studies, as most of them focus on the bigger player in trunk extension, the erector spinae muscle. The active exoskeleton JAP generates a lumbar traction, by applying forces to extend the exoskeleton’s interfaces at the pelvis (iliac crest) and the rib-cage (arcos costalis) (see Table 1). The area of the MF is below the supportive area of the exoskeleton. This might be a reason why JAP showed a significant main effect and higher MF activity compared to HTRI, HUN, and CRAY, even though a reduction in ES activity was found. It can be argued that the lumbar traction leads to a reduced spinal flexion in the supported area. Hence, similar to issues after a vertebral fusion surgery, increased loads and pressure on the intervertebral disks below the stiffened area of the spine can occur (Park et al., 2004).

#### 4.2.3. Hip extension support for lifting

The active exoskeleton CRAY exerts hip extension torque by two electrical rotatory motors positioned at the hip joint, and interfaces connected at the trunk in the form of a vest and at the upper thighs (see Table 1). In principle, the external force transmission should decrease the user’s hip and trunk extension torque, which could lead to the observed reductions in back muscle activity.

In this sense, less muscle activity in the back muscles does not necessarily imply a proportional reduction in mechanical load to the passive structures of the spine, which is one of the risk factors of low back pain. Additionally, as emphasized by McGill et al. (2003) a coactivation of a series of muscles surrounding the trunk in highly coordinated patterns is needed to assure and protect the integrity of the spine during heavy or repeated lifting tasks (McGill et al., 2003). Activity reduction in a specific muscle group or in parts of a kinetic chain can disturb these activation patterns and decrease trunk stability. Hence, a more comprehensive assessment (e.g., including simulation of joint loads, muscle synergy analysis, activation patterns, and trunk stability assessment) would be beneficial to correctly integrate the muscle activity reductions and find clearer indications about long-term injury mitigation.

### 4.3. Adaptations of lower-body kinematics

#### 4.3.1. Task completion time

The exoskeletons had an influence on the kinematics, especially during the lifting and lowering phase, by slowing down parts of the movement and in some cases even inhibiting end-range joint flexions. HTRI, HUN, and the active exoskeleton CRAY prolonged the lifting by 0.34–0.40s (11–13%). The two passive exoskeletons also increased the time to lower the box to the floor by 0.32–0.40s (10–13%). A substantially prolonged task duration was found for the active exoskeleton JAP. The time to lift the box increased by 0.92 s (30%) and lowering the box to the floor was also prolonged by 0.65 s (21%).

Literature is sparse on the exoskeletons’ effect on task duration or angular velocity. This might be due to the fact that mainly passive back-support exoskeletons were analyzed, and an effect on movement speed was rarely considered in that context (Hoffmann et al., 2022). However, our findings suggest that kinematic adaptions due to exoskeletons need to be analyzed in a broader sense. The prolonged task duration during lifting can be caused by additional loads appearing during bending down due to the elastic elements in the passive exoskeletons HUN and HTRI. For JAP, it seemed that the electrical motors of the actuators could not operate in line with the hip angular velocity of the users, leading to a slowed-down lowering and lifting motion. Maximum actuator speed needs to at least match the preferred kinematics of the users to allow free movement patterns. Reduction effects in mean activity of the back muscles must be interpreted in the light of a prolonged task duration, which might reduce or even diminish possible relieving effects of the device due to longer exposure of the loads to the body.

#### 4.3.2. Joint angle RoM

A statistical trend showed reduced hip RoM by 10° during phase 1 using JAP. RAK caused a reduction in knee RoM by 12°. HUN caused a 9° reduction in spinal flexion in the trunk during lifting from the floor. Trunk inclination was significantly increased by HUN and CRAY during phase 4 (lifting from table) and by almost 12° during phase 6 (lowering to floor).

Previous studies analyzing the effects of various passive exoskeletons on the RoM and kinematics of the trunk and lower body reported mixed results (Sadler et al., 2011; Koopman et al., 2019; Glitsch et al., 2020; Luger et al., 2021). On first impression, obviously, the exoskeleton must have a major impact on the affected kinematics, but literature shows that it is the interaction of exoskeleton and lifting style (whether it was instructed or intuitively adopted due to the use of the exoskeleton), which seems to have a larger impact on the kinematics of the lower body (Luger et al., 2021). During static forward bending, passive exoskeletons led to larger hip flexion angles (Bosch et al., 2016). Dynamic lifting, in general, showed a reduction in hip RoM in the exoskeleton condition (Glitsch et al., 2020). But, when stoop lifting was instructed, hip and ankle flexion increased during the use of the exoskeleton (Sadler et al., 2011; Luger et al., 2021). Additionally, a more rigid trunk was reported by Sadler et al. (2011), meaning less flexion in the spinal joints, which is in line with what we observed for HUN during lifting. This shows the interconnectivity of all joints within the kinetic chain and the motion redundancy when performing such a lifting task. As the task still needs to be fulfilled, an inhibition in some joints must be compensated by other joints to reach the desired depth to lift the object. JAP’s lower interface at the pelvis caused a mechanical restriction during pronounced hip flexion leading to the reduced RoM. A precise and adequate selection of exoskeleton size might reduce this effect in part. The interfaces of HUN and RAK are attached below the knees. The effect for HUN showed high interindividual variability in the kinematic adaption, but RAK’s effect was more homogenous among the subjects. The reduced knee RoM might be a result of adapted movement patterns along the kinetic chain, as no local mechanical flexion inhibition occurred at the joints.

#### 4.3.3. Lifting style

Although some studies allowed “freestyle” lifting with a preferred lifting pattern, most previous studies regulated the biomechanics of lifting by instructing the participants to lift the loads either with extended legs and a strong hip hinge (stoop lifting) or with bended knees (squat lifting) (Abdoli-E et al., 2006; Frost et al., 2009; Alemi et al., 2019; Glitsch et al., 2020). As an aim of this study was to maximize transferability of our results to a complex working environment, lifting style was not regulated in any form and participants have chosen to lift the box in an individually preferred movement.

Various authors mentioned the effect of changed movement patterns and lifting style, specifically trunk flexion and inclination, on back muscle activity and loads on the lumbar spine (de Looze et al., 2016; Zhang et al., 2016; Alemi et al., 2019; Koopman et al., 2019). These adaptions in movement pattern due to exoskeleton use can be highly individual (Theurel and Desbrosses, 2019), but might have a strong impact on the supportive effect of the devices, and should therefore be considered carefully during the introduction and application of exoskeletons in a workplace environment.

### 4.4. Limitations

The following limitations of the current study need to be addressed. The participants taking part in the study were all healthy, young, well-trained, and predominantly male (*n* female = 3). To improve general validity of our findings, a more heterogeneous sample including logistic workers of all ages would be beneficial. Regarding the non-standardized lifting style, two limiting factors must be considered. First, although a familiarization period with each exoskeleton was carried out, long-term use of a supportive device would lead to an individually preferred movement pattern, probably different to the movement observed during the experiment. Second, participants in an observed laboratory environment might tend to adapt their lifting style to meet prevalent expectations on supposedly correct behavior, especially in nonprofessional logistic workers with unstable lifting patterns (Sonderegger and Sauer, 2009).

Additionally, our findings are limited to short-term effects of exoskeleton use. As muscular fatigue plays a major role in overuse syndromes and injuries, further studies need to investigate long-term adaptions over the period of up to a full work shift. The analysis of RoM changes in the main joints of the lower body indicated high interindividual variability in the adaption of movement patterns due to the use of different exoskeletons. Adaptions seemed to scatter in different directions between participants, hence, a significant effect was only found for homogeneous inhibitions. A comprehensive analysis of whole-body movements should be conducted to clarify interaction effects of the exoskeletons with preferred movement patterns instead of single-joint analysis of the kinematics.

## 5. Conclusion

The study evaluated the acute biomechanical effects of exoskeletons with different functional support mechanisms. We were able to show intermuscular differences in the activity reduction between the exoskeletons, especially when the functional mechanism and the designed force path differed substantially. In general, a reduction in lower and upper back muscle activity was found in four of the observed exoskeletons. Reduction effects were primarily found during the phases of lifting and lowering the object (to ground level). A decrease in muscle activity alone cannot tell the full story regarding the reduction of strain and stress on passive structures. Passive exoskeletons with elastic elements along the posterior side of the body might only replace contractile force of the muscles without relieving effects on the passive structures of the spine. Exoskeletons should be assessed based on their functional mechanisms and the designed force path to correctly interpret the supportive effect for long-term injury mitigation and prevention. Effects on the joint RoM were found to be small for most exoskeletons in this study. Nevertheless, trunk flexion and inclination were affected by HUN and CRAY resulting in a more rigid inclined upper body. Hence, interference effects of the devices with preferred movement patterns of the user and possible side effects due to that should be analyzed more precisely. Further, comprehensive movement analysis with an evaluation of joint torques and loads should be conducted in rested and fatigued state to assess mid- to long-term injury preventive effects of exoskeletons in real workplace environments.

## Data Availability

The data that support the findings of this study are available on request from the corresponding author, B.R. The data are not publicly available due to privacy protection regulations, for example, their containing information that could compromise the privacy of research participants.
